# Past Variance and Future Projections of the Environmental Conditions Driving Western U.S. Summertime Wildfire Burn Area

**DOI:** 10.1029/2020EF001645

**Published:** 2021-02-06

**Authors:** Steven J. Brey, Elizabeth A. Barnes, Jeffrey R. Pierce, Abigail L. S. Swann, Emily V. Fischer

**Affiliations:** ^1^ Department of Atmospheric Science Colorado State University Fort Collins CO USA; ^2^ Department of Atmospheric Science University of Washington Seattle WA USA

**Keywords:** wildfire, burn, area, west, climate, change

## Abstract

Increases in vapor pressure deficit (VPD) have been hypothesized as the primary driver of future fire changes. The Coupled Model Intercomparison Project Phase 5 (CMIP5) models agree that western U.S. surface temperatures and associated dryness of air as defined by the VPD will increase in the 21st century for Representative Concentration Pathways (RCPs) 4.5 and 8.5. However, we find that averaged over seasonal and regional scales, other environmental variables demonstrated to be relevant to flammability, moisture abundances, and aridity—such as precipitation, evaporation, relative humidity, root zone soil moisture, and wind speed—can be used to explain observed variance in wildfire burn area as well or better than VPD. However, the magnitude and sign of the change of these variables in the 21st century are less certain than the predicted changes in VPD. Our work demonstrates that when objectively selecting environmental variables to maximize predictive skill of linear regressions (minimize square error on unseen data) VPD is not always selected and when it is not, the magnitude of future increases in burn area becomes less certain. Hence, this work shows that future burn area predictions are sensitive to what environmental predictors are chosen to drive burn area.

## Key Points

- Climate models agree that vapor pressure deficit will increase throughout the western United States
- Vapor pressure deficit is not required to explain observed variability in past summer wildfire burn area for all western U.S. ecoregions
- Future wildfire burn area estimates are highest in regions where vapor pressure deficit explains substantial past variance in burn area

## 1 Introduction

Smoke from western United States (U.S.) wildfires can impact atmospheric composition on local, synoptic, and continental scales (Baker et al., 2016; Brey et al., 2018; Brey & Fischer, 2016; Ford et al., 2017; Lassman et al., 2017; Saide et al., 2015; Val Martin et al., 2015; Wiedinmyer et al., 2006). The air quality impacts of wildfire smoke can be especially pronounced in the western United States (McClure & Jaffe, 2018b; O'Dell et al., 2019), where the majority of particulate matter with diameters less than 2.5 μm (PM_2.5_) National Ambient Air Quality Standards (NAAQS) exceedances can be attributed to wildfire smoke (Liu et al., 2016). A growing body of research has demonstrated that ozone, a gas phase criteria pollutant in the United States, can also be enhanced in the presence of wildfire smoke (Brey & Fischer, 2016; Jaffe et al., 2013; Lu et al., 2016; McClure & Jaffe, 2018a). The deteriorated air quality due to wildfire smoke can impact public health on large scales (Delfino et al., 2009; Fisk & Chan, 2017; Gan et al., 2017; Koman et al., 2019; Lipner et al., 2019; Rappold et al., 2011). Given this suite of impacts on air quality, alongside other impacts on people, property, and economics, there is a need to understand (1) what drives year‐to‐year wildfire activity, (2) how those drivers may change in the future, and (3) how future burn area and total emissions may respond. Many factors contribute to variations in western U.S. wildfire activity including natural climate variability, climate change, and land management (Abatzoglou & Williams, 2016; Pechony & Shindell, 2010; Short, 2015; Westerling et al., 2006, 2014). Variability in long‐term climate and year‐to‐year weather can influence the availability and flammability of fuel (Littell et al., 2009, 2016).

Statistical models are one tool used to explain observed variance and/or predict future fire activity, and they often leverage correlations between wildfire burn area and environmental variables influenced by meteorology. Many studies that leverage these observed relationships predict significant increases in western U.S. fire activity under climate change (e.g., Flannigan et al., 2009; Liu et al., 2016; McKenzie et al., 2004; Spracklen et al., 2009; Westerling et al., 2011; Yue et al., 2013). On seasonal‐to‐annual timescales, western U.S. wildfire activity (e.g., burn area) can be significantly correlated with temperature, vapor pressure deficit (VPD), hot‐dry‐windy days, soil moisture, relative humidity, and precipitation (Abatzoglou & Williams, 2016; Brey et al., 2018; Forkel et al., 2017; Kloster & Lasslop, 2017; Littell et al., 2009; Williams et al., 2012, 2017; Parks et al., 2018; Pechony & Shindell, 2010; Rothermel, 1983; Short, 2015; Srock et al., 2018; Westerling et al., 2006, 2014; Yue et al., 2013). Different environmental variables have been shown to be important in different regions of the western United States. For example, wildfire burn area in the southwest United States has been shown to be related to drought at varying lagging timescales (Littell et al., 2009; Williams et al., 2015).

Studies have shown that western U.S. wildfire burn area has increased since the 1980s as the western United States has warmed (e.g., Westerling et al., 2006). From an energy perspective, temperature alone is not an ideal measure of wildfire potential. At the scale of individual wildfires, fire spread is a series of ignitions where heat from the fire raises fuels to ignition temperature (Rothermel, 1983; Simms & Law, 1967). Before ignition of fuel can occur, water must be evaporated from the fuel, which comes at the cost of the latent heat of vaporization (2,257 J/g); thus, the flammability and rate of fire spread depends on fuel‐moisture content (Bradshaw et al., 1984; Fosberg et al., 1971; Rothermel, 1972; Simms & Law, 1967; Stocks et al., 1989). The physical link between temperature and fuel flammability is better represented by atmospheric VPD. Dead fuels, such as litter, passively respond to ambient atmospheric conditions such as VPD. Thus, atmospheric VPD has widely been considered the aridity metric that increasing temperature works through to dry fuels (e.g., Anderson & Rothermel, 1965; Rothermel, 1983; Williams et al., 2015), and as a result, much recent work in this field focuses on changes in VPD as opposed to changes in temperature and other environmental predictors.

The overarching goal of this work is to understand how changing environmental conditions contribute to the uncertainty of 21st century western U.S. wildfire activity quantified by burn area, a metric proportional but not perfectly correlated to smoke air quality impacts. Previous studies have shown there are many factors that contribute to this uncertainty (e.g., emission factors, changing wildland‐urban interface, sources and abundances of ignition sources, and changing fuel loads). Our work examines the environmental conditions (wind speed, precipitation, RH, VPD, and root zone soil moisture) that contribute to future burn area estimates, and we compare scenario and intermodel spread in these conditions across three mountainous western U.S. ecoregions. These three ecoregions contain much of the forested mountains of the western United States, the highest carbon emission factors, and greatest fuel loadings in the region (kg‐fuel m^−2^) (Abatzoglou et al., 2017; Urbanski et al., 2018; van der Werf et al., 2010). These ecoregions were the largest emitters of smoke PM_2.5_ in the western United States between 2003 and 2015 (Urbanski et al., 2018). Our methods do not account for changes in fuel loading, so we continue our analysis motivated by the assumption that these regions will continue to be characterized by large fuel loading and emission factors in future decades.

For this work we use Lasso regression to objectively identify the antecedent and concurrent set of environmental conditions that best explain observed variance in interannual summertime (June, July, and August, JJA) large wildfire (>1,000 acres) burn area for western U.S. ecoregions. We focus on JJA as these months have produced the greatest burn area in recent decades (Brey et al., 2018). This is done individually for each ecoregion to allow for varying relationships between burn area and environmental conditions. We use the Coupled Model Intercomparison Project Phase 5 (CMIP5) (Taylor et al., 2012) model output to quantify the mean change and future spread in the environmental variables that best explain historical variance for each fire‐prone ecoregion. We contrast the trends and spread between models made for each candidate variable and discuss what the changes may imply for future burn area. Finally, we combine historical relationships between burn area and select candidate variables with CMIP5 output to examine the spread in future burn area as forecast by the objectively selected variables. This work shows that the predicted change in burn area is extremely sensitive to what environmental predictors are chosen to drive burn area.

## 2 Data and Methods

### 2.1 Selection of Candidate Variables

We define environmental conditions previously identified to be important for wildfire occurrence as “candidate variables.” The candidate variables used in this work include wind speed, precipitation, RH, VPD, and root zone soil moisture. These variables (1) have been demonstrated to impact wildfire occurrence by other studies and/or (2) are related to or are an indicator of the moisture budgets that are relevant for fuel availability, flammability, or plant stress, and (3) are available in both historical reanalysis datasets and CMIP5 future‐projection simulation output, which enables the training of statistical models from historical information and use of these models for forward projections. VPD and RH are correlated and both related to aridity and temperature. We choose to include both because RH is tightly coupled to fire weather, even at low temperatures (Srock et al., 2018; Yue et al., 2013). For a given ecoregion, we objectively select the variables that best explain observed variability using Lasso regression (section 2.5). This approach allows for us to learn from the data rather than confirm a hypothesis of a specific variable's importance.

### 2.2 ECMWF ERA‐Interim Reanalysis

For historical data on environmental variables, we use European Center for Medium‐Range Weather Forecasts (ECMWF) ERA‐Interim monthly reanalysis fields (followed by ERA‐Interim specific abbreviations) (Dee et al., 2011), total precipitation (tp), wind speed (si10), relative humidity, VPD, both (calculated using dew point temperature, surface pressure, and Tetens formula), and soil moisture (mrso). All data were downloaded on 0.75° × 0.75° grids. To make the ERA‐Interim output directly comparable to CMIP5 output (described next), variables were bilinearly regridded to a common grid with 2° × 2.5° degree grid spacing (Schulzweida, 2019). Where necessary, ERA‐Interim units were converted to match the units used by CMIP5 models. A total root zone (~3 m depth) soil moisture field was created by converting volumetric soil water in Layers 1 through 4 (total depth of 2.89 m) to kg m^−2^ by assuming the density of all water content to be 1,000 kg m^−3^.

### 2.3 CMIP5

To estimate the future change and spread in wildfire‐relevant environmental predictors, we used output from the Phase 5 of the CMIP5 (Taylor et al., 2012). We used the following monthly variables (followed by CMIP5 specific abbreviations): near‐surface temperature (tas), precipitation (pr), Near‐Surface Wind Speed (sfcWind), near‐surface relative humidity (hurs), and soil moisture content (mrlsl). VPD was estimated using mean temperature and relative humidity via Tetens formula. We note that there is an alternative way of calculating VPD using maximum and minimum temperature. This method can better account for the nonlinear response of VPD to temperature (Ficklin & Novick, 2017). However, ERA‐interim does not explicitly provide minimum and maximum temperature in a way that would be straightforward to compare to CMIP5 output. In order to use a single data source for historical conditions that can most easily be compared to CMIP5 output, we chose to use mean temperature instead.

Intermodel spread between models was quantified using the r1i1p1 ensemble member for each climate model. We were interested in quantifying intermodel spread and scenario spread, which can be accomplished with a single ensemble member. Though not as comprehensive as using multiple ensemble members, the use of a single ensemble member still allows our methods to account for internal variability of the climate system. Scenario spread is quantified by examining output from Representative Concentration Pathways (RCP) 4.5 and 8.5, scenarios that correspond to different greenhouse gas climate forcing (W m^−2^) scenarios for the 21st century (van Vuuren et al., 2011). RCP 4.5 represents an emission scenario where greenhouse gas emissions begin to slow in the mid‐21st century, RCP 8.5 represents a scenario where emissions continue to grow throughout. In order to bias correct CMIP5 data using the reanalysis data, historical (pre‐2006) CMIP5 data were also downloaded. The data from all models were regridded to the GFDL‐CM3 2° × 2.5° degree grid using bilinear interpolation for atmospheric domain variables and distance‐weighted average remapping for land domain variables (Schulzweida, 2019). Data for this work were downloaded from the Earth System Grid Federation (https://esgf-node.llnl.gov/search/cmip5/). The CMIP5 models used in the results section are ACCESS1‐0, ACCESS1‐3, CNRM‐CM5, CanESM2, GFDL‐CM3, GFDL‐ESM 2G, GFDL‐ESM 2M, GISS‐E2‐H, GISS‐E2‐H‐CC, GISS‐E2‐R, GISS‐E2‐R‐CC, MIROC‐ESM, MIROC‐ESM‐CHEM, MIROC5, and INMCM4 (Ackerley & Dommenget, 2016; Chylek et al., 2011; Dunne et al., 2012; Griffies et al., 2011; Schmidt et al., 2014; Voldoire et al., 2013; Volodin et al., 2010; Watanabe et al., 2010, 2011). These models were selected based on data availability for the time span of interest (1983–2099), RCP 4.5, RCP 8.5, availability for download at the time of this analysis, and availability of the required environmental variables.

#### 2.3.1 CMIP5 Soil Moisture Data

While accurate soil properties are required to accurately simulate soil‐water content and implications for seasonal drought stress (Peterman et al., 2014) the soil schemes in the CMIP5 models vary substantially (Berg et al., 2017). For example, the active soil depth varies between 3 and 42 m. Soil moisture by layer (mrlsl) in the CMIP5 archives include water in all phases. The different active soil moisture depths make intermodel comparison of total soil moisture impractical, so instead, we regrid the soil moisture to include only the top 2.89 m, which is the part of the soil column most accessible to ecosystems (root zone soil moisture, regridded down to 2.89 m to match ERA‐Interim soil moisture depth); similar approaches have been used to compare models in other studies (e.g., Berg et al., 2017). A depth of 2.89 is close to the maximum soil depth of several models, which is at a minimum 3 m. The hydraulic properties of soils will also vary among models, which will partially contribute to differences seen between them. The vertical interpolation is required due to the significantly varying depths and layers output in CMIP5 coupled models. Due to the different ways CMIP5 models represent soil moisture processes, integrated water in the top ~3 m does not have the same meaning in terms of drought stress across models.

### 2.4 MTBS

This work quantifies interannual variability of wildfire burn area using the wildfires documented in the Monitoring Trends in Burn Severity database (MTBS). MTBS is a multiagency program with the goal of consistently mapping the burn area and severity of large wildfires in the United States in order to monitor the effectiveness of the National Fire Plan and Healthy Forests Restoration Act (Eidenshink et al., 2007). These data include wildfire burn area for the western United States between 1984 and 2016 for wildfires larger than 1,000 acres (404 ha) (Eidenshink et al., 2007). Wildfires larger than 1,000 acres account for the majority of burn area in the western United States (Brey et al., 2018). MTBS uses the Normalized Burn Ratio (dNBR) from Landsat to detect fire burn scars and severity (Eidenshink et al., 2007). MTBS data are largely divided into mapping zones based on Bailey's Ecological Sections (Bailey, 1983), but this mapping is slightly altered to account for meaningful administrative boundaries (Eidenshink et al., 2007). Though MTBS assesses the severity of burn area, our work makes no distinction between severities to create an aggregate burn area. MTBS will generally report less burn area than totals tabulated by incident reports (Eidenshink et al., 2007). For example, in 2004 MTBS mapped 7,781,049 acres of wildfires while statistics compiled by the National Interagency Coordination Center (NICC) documented 8,097,880 acres (Eidenshink et al., 2007). MTBS maps wildfires and prescribed (Rx) fires. Our work aims to focus on the climate and biophysical controls on fire occurrence, so we exclude Rx fires from our analysis, and from here forward we refer to the remaining types in MTBS as “wildfires” (similar to Finco et al., 2012). One of the fire types in MTBS is “unknown.” There is a possibility that some of these fires were prescribed; however, MTBS documents only one documented Rx fire in the western United States that occurs in the months June, July, or August, the months of interest in this analysis, so we expect prescribed fires to make up a small amount of the burn area contributed by the unknown fire type category. The 33 years of wildfire data in MTBS provide a longer time period than other incident based datasets that include additional small wildfires (e.g., FPA FOD developed by Short, 2014), which grants analysis of trends and variability in wildfire occurrence additional validity. MTBS data were downloaded as Fire Occurrence Data (point locations rather than burn scar shapes; from https://www.mtbs.gov/direct-download). These fire data are then assigned to Baileys divisions to create monthly burn area totals aggregated by areas with similar geographic and ecological characteristics.

### 2.5 Bailey's Ecoregions

**Figure 1:**
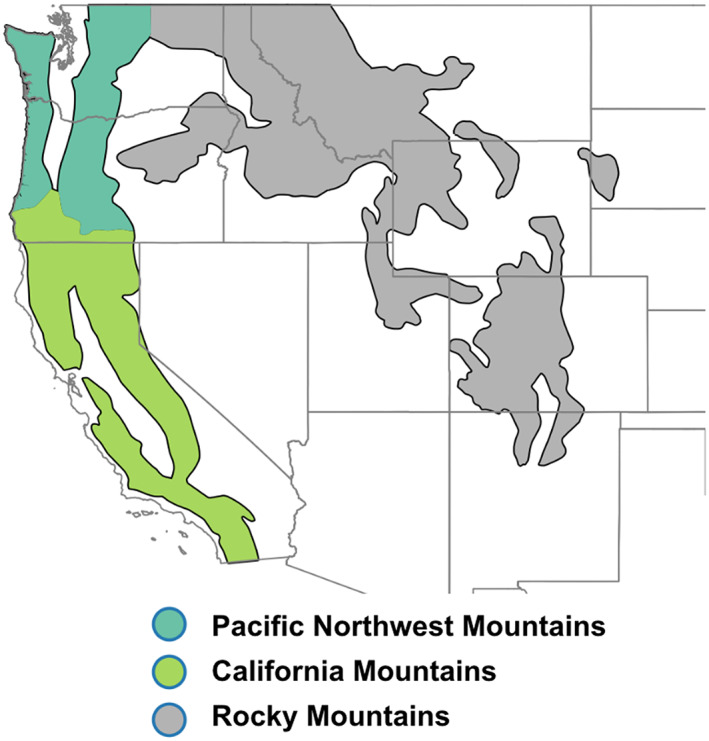
The western U.S. Bailey's divisions analyzed in this work (hereafter referred to as ecoregions). The Pacific Northwest Mountains (officially named Marine Regime Mountains) in this work excludes portions of that region above 50°N.

To account for how environmental drivers of wildfires may differ between regions, we aggregate MTBS burn area data, ERA‐Interim reanalysis, and CMIP5 model output by Bailey's ecoregions. Bailey's ecoregions include four levels, with each subsequent level containing smaller, more narrowly defined ecosystems. We choose to use the “divisions” level, a subset of the “domain” (most coarse) level. These nearly contiguous divisions allow us to aggregate wildfires that occur in areas of similar temperature and precipitation. The Temperate Steppe, Marine, and Mediterranean Regime Mountain divisions (hereafter referred to as Rocky Mountains, California Mountains, and Pacific Northwest Mountains respectively) (shown in Figure 1) contain much of the forested mountains of the western United States (Abatzoglou & Williams, 2016), and correspondingly, some of the largest and most destructive wildfires, as well as the highest PM_2.5_ emissions between 2003 and 2015 (Urbanski et al., 2018). Thus, our analysis focuses on these three ecoregions.

Baileys division data were downloaded as shapefiles from https://www.fs.fed.us/rm/ecoregions/products/map-ecoregions-united-states/# website. The divisions (hereafter referred to as “ecoregions”) used in this work are shown in Figure 1.

We recommend caution looking at any of these regions as homogenous, as there is considerable subregion variability in topography, precipitation, temperature, population density, and flammable fuel coverage in each of these regions. Thus, the mean environmental conditions in these regions are a somewhat crude representation of the environmental conditions corresponding to both historical and future wildfires within these ecoregions. However, given the future looking perspective of this paper, which relies on using global climate model output, this is an acceptable weakness, as climate models are unable to represent environmental conditions at scales fine enough to relinquish this concern.

### 2.6 Wildfire Predictor Identification and Regularization With Lasso Regression

We use the Least Absolute Shrinkage and Selection Operator (hereafter Lasso) regression to create regularized linear models for each of the three ecoregions in the western United States shown in Figure 1. Lasso is a regression method that performs regularization and variable selection (Tibshirani, 1996). The Lasso was developed to increase the prediction accuracy of ordinary least squares (OLS) regression by reducing the variance of predicted values (Tibshirani, 1996). The Lasso accomplishes high prediction accuracy and lower variance through a constraint on the sum of the absolute values of the model parameters, which results in some predictor coefficients being set to exactly 0. When the regularization term α is 0, the Lasso is the same as OLS. The higher the value of α, the more regularized (simpler the model is forced to be to avoid overfitting) the regression model becomes, and more predictors coefficients are set to exactly 0. Lasso has been demonstrated to make a good compromise between model complexity and model performance (Loukina et al., 2015; Pedregosa et al., 2011). The Lasso can select multiple correlated predictors. For example, if dry spring seasons are correlated to dry summer seasons, and both are good predictors of wildfires, using both gives the desired insights and variance explained needed for this work.

In our use of Lasso, environmental variables from ERA‐Interim reanalysis are used as predictors or “features.” ERA‐Interim data are spatially subset to include only the grid boxes that overlap a given ecoregion's geographic extent. This results in larger ecoregions being represented by more grid boxes than smaller ecoregions. We take the temporal average of overlapping features for three seasons, winter (months November, December, January, and February), spring (months March, April, and May), and summer (months June, July, and August). This temporal averaging is a necessary step to allow for lagging relationships between environmental variables and summer burn area observed in other studies to be accounted for in this analysis.

The underlying assumption of these methods is that year‐to‐year variability relationships can be leveraged to estimate long‐term changes in burn area. Due to the limited quantity of wildfire data, we set up our regression to explain variance in year‐to‐year wildfire burn area, rather than the trend in environmental variables or wildfire burn area. To avoid a spurious model where the target function and predictors are well correlated because of a shared trend, we detrend the environmental variables and the burn area time series before fitting the regression. This ensures that the regression predicts *year‐to‐year* variability and that correlations between burned area and features are due to interannual covariability. Regressions are performed independently for each ecoregion. Similar to other work (e.g., Littell et al., 2009; Williams et al., 2015), the summer burned area was log_10_ transformed to account for the exponential distribution of annual burned area and to mitigate heteroscedasticity. Heteroscedasticity results from year‐to‐year quantities that span orders of magnitude, increasing over time, so the variance is not stationary. Without taking the log_10_ transformation of summer burn area, some ecoregions exhibited upward trends in model residuals. We observe higher linear model performance (*R* ^2^ values) when summer burn area is log_10_ transformed. Because the log_10_ of zero is undefined, we set years with zero acres of burn area to 1. This only occurs in the Pacific Northwest Mountains. In order to make comparing environmental variances with different units easier, we standardize each feature ( *μ* = 0, *σ* = 1) for the historical training period (1984–2016) such that the units of model coefficients are the same (log_10_(burn area) *σ* ^−1^).

### 2.7 Quantification of Spread in Key Wildfire Predictors

We use CMIP5 model output to show how environmental variables (i.e., features) selected by Lasso regression may change in the future. We examine changes in year‐to‐year variability as well as changes due to long‐term trends. We bias correct CMIP5 modeled historical and future projections interannual standard deviation and mean value between 1984 and 2016 to match those estimated by the ERA‐Interim reanalysis fields. This was done for each feature predicted by each CMIP5 model individually using the following procedures. (1) The linear trend is removed from the 1984–2016 CMIP5 feature times series (e.g., summer precipitation for model ACCESS1‐0). (2) The standard deviation, which after detrending is due entirely to year‐to‐year variability, is calculated for the detrended 1984–2016 time series. (3) The detrended 1984–2100 CMIP5 time series is multiplied by the ratio of the detrended ERA‐Interim feature standard deviation over the detrended CMIP5 feature 1984–2016 standard deviation. At the completion of this step, the CMIP5 feature standard deviation for 1984–2016 matches ERA‐Interim. (4) The 1984–2016 linear trend is added back to the CMIP5 feature. (5) To correct the CMIP5 model bias offset, the mean value of the time series from 1984–2016 is replaced by the ERA‐Interim mean for the same years.

Differences in modeled and observed standard deviations and trends suggest that climate models are not simulating what is estimated by ERA‐Interim. Scaling these data can also inflate confidence in models as they have been forced to look more similar than the original output. However, we choose to scale the variance in this work because it eases the interpretation of the changes in environmental predictor values in the following ways. (1) It allows for a straightforward interpretation of changes observed by models from the historical period to the simulated future. (2) It gives us the ability to observe how the spread in a variable changes due to changes in the range of year‐to‐year values. (3) It ensures the spread in the estimates of future wildfire activity estimated by CMIP5 output using the regression models is not artificially narrow due to a small variance in CMIP5 model variables. Finally, bias‐corrected CMIP5 features are standardized in the same way as the ERA‐Interim features used to train the Lasso, by subtracting the 1984–2016 ERA‐Interim mean and dividing by the standard deviation. This adds additional interpretability to the results. For example, if a CMIP5 model feature value for summer precipitation in the year 2020 was 0, that would mean that CMIP5 model simulated summer 2020 precipitation to be the same as the mean historical value.

## 3 Results

### 3.1 Historical Relationships Between Wildfire Burn Area and Predictors

**Figure 2:**
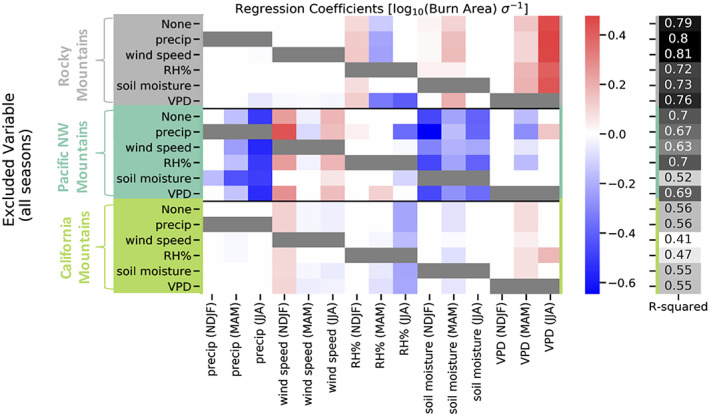
Each row shows the value of the coefficients (color) for different Lasso regression models fit in each of the three ecoregions in this work (indicated by the colors surrounding the excluded variables on the vertical axes). Within a given ecoregion, the rows correspond to the environmental variable (all seasons) that were not included in fitting each Lasso regression model. Coefficient values for variables not included are shaded dark gray. Each Lasso regression model was fit after tuning α (regularization term) via leave‐one‐out (year) cross validation. Ecoregions are denoted and separated by the colored horizontal lines. The corresponding regression *R*‐squared values are shown in the 2right panel.

Figure 2 shows the regression coefficients and *R*‐squared values for Lasso regression models fit to predict burn area using various combinations of environmental variables as predictors. For the models shown in Figure 2, the model complexity parameter (*α*) was tuned using leave‐one‐out cross validation. Figure 2 shows how the best fit coefficients change for each region when the model is restricted from using a given predictor for all seasons. The colors in Figure 2 represent the magnitude of coefficients for the regression fits. For a given row, the variables that are grayed out are the variables not used in the fitting (there are no grayed out variables in the “None” rows, where all available variables were used). Near‐white hues indicate that a given coefficient is either very small or exactly zero. The larger the absolute value of the coefficient, the larger impact that variable has on predicted burn area per unit change of that predictor. Coefficients can be compared across ecoregions, and many variables have coefficients exactly equal to 0 (white), indicating that the linear model with the most skill (highest *R*‐squared) usually only needs a small subset of the available predictors (e.g., Rocky Mountain region regressions). Most features have the same sign across all ecoregions where they are used, indicating a consistent regression relationship between environmental variables and burn area across ecoregions and predictor sets.

There are several notable relationships that emerge from Figure 2. Summertime VPD is an important predictor for historical wildfire burn area in the Rocky Mountains. By contrast, there are a number of variables that contribute to regression predictions of wildfire burn area in the Pacific Northwest. Soil moisture, precipitation, and wind speed stand out for this region, while VPD is only nonzero when precipitation is excluded. Weak correlations between gridded wildfire and wind data have been observed in other studies (e.g., Brey et al., 2018; Crevoisier et al., 2007; Yue et al., 2013). Thus, the importance of wind speed in the Northwest is surprising. It is possible that wind speed is acting as a proxy for other wildfire‐relevant conditions, like the location of seasonal storm tracks or the frequency of strong cyclonic flow. For the California Mountains, the light shading indicates that the coefficients are ubiquitously small. The coefficient for summer VPD is only appreciable when RH is excluded. An additional pattern to note in Figure 2 is that as variables are left out of the Lasso regression fitting, feature coefficients change in magnitude but rarely sign, an indicator of the robustness of the underlying physical relationships. Figure 2 also provides the *R* ^2^ value for each regression model (which are differentiated by the variables included). These *R* ^2^ would be even higher if using OLS regression, which does not include any kind of regularization. Due to the use of Lasso regression, the fits performed with all of the available predictors have similar *R* ^2^ values to the regressions with access to fewer models, evidence that overfitting is avoided. The highest R^2^ value is achieved in the Rocky Mountains for the regression excluding wind speed, though this model has similar *R* ^2^ and coefficients to estimates made with all variables and those made excluding precipitation.

Model sensitivity to available data was explored excluding both variables (as shown in Figure 2) and years of data. For simplicity, we present the results for regression models that left out a predictor here. The results from regression models that left out years (34 total regression models per ecoregion) are provided in the supplement as it is much more challenging to show.

### 3.2 Spread in Wildfire Predictors and Influence on Future Burn Area Projections

**Figure 3:**
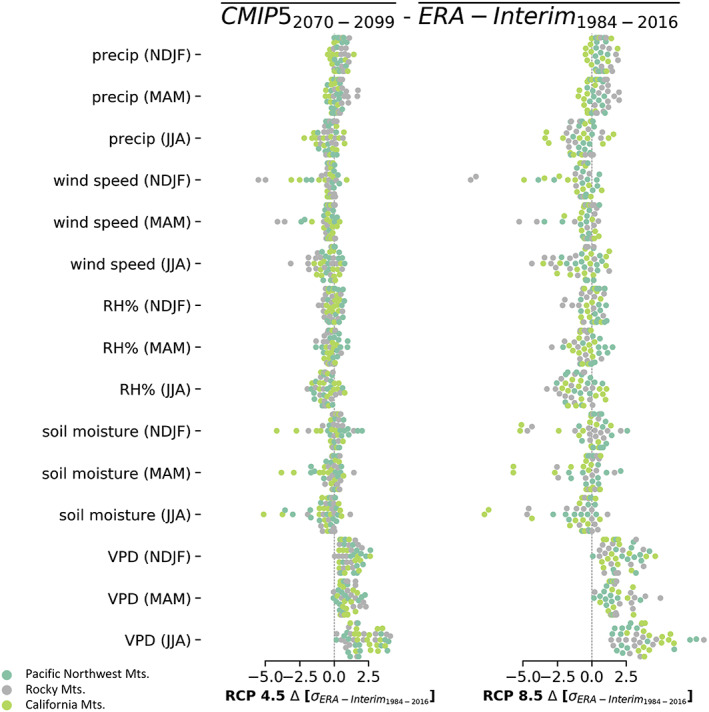
The change in environmental variable mean value (horizontal axis) from 1984–2016 to 2070–2099 shown for different features (rows) and ecoregions (colors). Each dot shows this change estimated by a single CMIP5 model for RCP 4.5 (left) and RCP 8.5 (right). The change in feature value is expressed in units of historical *σ* as estimated by detrended ERA‐Interim feature values from 1984–2016. Values that fall on the vertical line are features where the multidecade mean value has not changed between the two time periods. For a given ecoregion (color), the numbers of CMIP5 models (dots) is consistent but can differ between ecoregions.

Next we focus on how candidate environmental variables are predicted to change as estimated by CMIP5 models. Figure 3 shows how the long‐term changes simulated for different variables vary between ecoregions. Every CMIP5 model for both RCP scenarios show an increase in the winter, spring, and summer VPD. The spread in VPD is comparable to other features, but it is unique in that the estimates show only increases, across all CMIP5 models, RCP scenarios, and ecoregions. This is because increases in VPD are largely a function of increases in temperature, which is projected to increase nearly everywhere (Scheff & Frierson, 2013). In contrast, CMIP5 models show both increases and decreases in every other predictor for both RCPs. Most CMIP5 models show a decrease in summer precipitation in the Pacific Northwest, the variable with the largest coefficient absolute value in that region. From here forward, we only show results for RCP 8.5 because the changes are bigger.

### 3.3 Projected Wildfire Burn Area Using Historical Relationships

**Figure 4:**
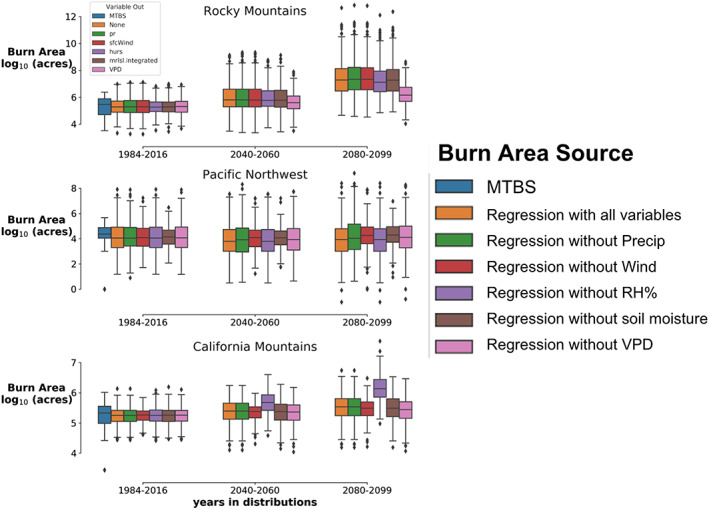
Each boxplot summarizes the distribution of summer (JJA) burn area that was observed (historical MTBS values) or estimated by linear models and CMIP5 output. Each boxplot shows these distributions for different spans of years (horizontal axis) predicted by different linear models (colors) driven by 15 CMIP5 model estimates of selected environmental conditions. For the non‐MTBS boxplots, the colors show which variable was excluded from the linear model used to estimate the burn area shown. Coefficients for these variables can be seen in Figure 2, and CMIP5 model changes can be seen in Figure 3.

This section compares the spread in future burn area for each of the Lasso regression models shown in Figure 2. Each regression model is fit with varying subsets of the available environment variables. For each of these regression models, an estimate of future burn area is calculated for each CMIP5 model independently, so each CMIP5 model is translated into an estimate of summer burn area for all years between 1984 and 2099. Figure 4 shows the CMIP5 ensemble median and spread of burn area estimated by each of these combinations aggregated over decades of interest.

In the California Mountains, when RH% is excluded as a predictor, the coefficient for summer VPD becomes nonzero (Figure 2). This trade‐off is not surprising because these variables are correlated and the Lasso tends to balance the use of each. However, this illustrates an important point. Despite the correlation between VPD and RH%, much larger future burn area estimates are predicted for the California Mountains for regressions that use VPD instead of RH% (Figure 4, bottom row purple boxes) because in units of historical variability, VPD increases much more than RH% throughout the 21st century (Figure 3). VPD driven increases in future burn area estimates are associated with the Rocky Mountain region as well. Here the regression coefficient for VPD is large whenever it is available (Figure 2), VPD increases in the future (Figure 3), and estimates that exclude VPD have much lower future burn area (Figure 4 top row, pink boxplot). In the Pacific Northwest Mountains, VPD is only used when precipitation is excluded and the magnitude of its coefficient is small compared to other variables (Figure 2). Thus, the future burn area distributions in the Pacific Northwest are nearly independent of VPD and in contrast to the other regions, the changes in burn area are much more consistent across the regressions (boxplot colors) and decades (boxplot horizontal axis). Figure 4 shows that estimates of future wildfire burn area are somewhat insensitive to what combination of environmental predictors and global climate models are used to make the estimate. The glaring exception is VPD. Future burn area estimates are larger wherever and whenever estimates rely more heavily on changes in VPD.

The spread shown by the boxplots in Figure 4 is likely an underestimate, as the linear models do not perfectly explain historical variability, and thus likely underestimate the true variability in the system. Even if our regressions had *R*_2_ values equal to 1, the spread displayed by the boxplots in Figure 4 would be an underestimate because our limited data, covering only 1984 through 2016, do not sample the full range of possible values (phase space) for environmental conditions or summer (JJA) burn area. CMIP5 models offer a more comprehensive sample of the environmental phase space. Combined, the CMIP5 models provide many more years of what environmental conditions could look like. This is how the CMIP5 driven burn area distributions can be wider than the observations distribution while still underestimating total spread. This is shown by the boxplots in Figure 4, which show the distribution of summer (JJA) burn area for MTBS and CMIP5 driven regression estimates for the years 1984–2016. The CMIP5 burn area distributions can also be narrower than the observations. This is most likely when the regression explains a small proportion of the historical variance. Thus, the spread for CMIP5 driven summer burn area for all regressions for the decades 2040–2060 and 2080–2099 shown in Figure 4 are more narrow than would be the case if our historical data covered the full phase space.

## 4 Discussion

One of the weaknesses of leveraging statistical models for predicting future wildfire burn area is the assumption that relationships between environmental conditions and burn area will remain stationary. Large‐scale land‐change disturbances and climate change are likely to alter the abundance and types of fuel available to burn in the west (Harris et al., 2016; Murph & Mooney, 2019; Parks et al., 2018; Williams et al., 2012). There is evidence that wildfire‐climate dynamics are likely to change as well (Higuera et al., 2015; Littell et al., 2016). For example, in the past, western U.S. forest wildfire activity has been flammability limited (Abatzoglou & Williams, 2016); however, ever increasing burn area would eventually result in limited fuel availability. In addition, due to internal variability of the climate system and anthropogenic‐caused climate change, the climate of the 21st century will include mean and extreme states of environmental variables that were not observed in the historical period (Collins et al., 2013; Mora et al., 2013), resulting in historical models extrapolating beyond the data they were trained on. There is also evidence that plant physiology responses to increased CO_2_ concentrations may reduce live fuel stress and flamaility. Plants absorb CO_2_ through stomata in their leaves and lose water to the atmosphere through the same pathway (Swann et al., 2016). Some plants lose less water per unit of carbon gain when CO_2_ mixing ratios increase because the gradient of CO_2_ between the leaf and atmosphere is reduced (Cowan, 1978). However, plant water use efficiency will only increase if the loss of water per unit of carbon gained is not offset by an increase in leaf area (Field et al., 1995). Assuming nearly constant leaf area, plant increased water use efficiency could result in reduced transpiration and increased soil moisture (Field et al., 1995). Combined, these changes could reduce plant water stress, even during droughts. Some observations show a decrease in transpiration due to increased water use efficiency (WUE) with increasing CO_2_ mixing ratios (Keenan et al., 2013; Peñuelas et al., 2011; van der Sleen et al., 2014; Warren et al., 2011), though the number of plant species this applies to and limits are not fully understood (Battipaglia et al., 2013). In cases where increased water use efficiency promotes increases in above‐ground biomass (Donohue et al., 2013), then the addition of fuels due to CO_2_ fertilization could further promote wildfire.

A second major limitation of this work is that burn area interannual variability may not scale with climate change the way it has in the historical period. The historical period considered in this work covered a time period where there were increases in burn area and VPD. Extrapolating these trends into the future would result in different future burn area estimates than our cross‐validated interannual variability based approach. The climate models and regional scales used in this work are only able to capture changes at large scales; however, there are subsets within western U.S. ecoregions that may in fact cool (reduced VPD) in the future as there is a decoupling of scales due to topography (Daly et al., 2009). This work uses a single CMIP5 ensemble member (r1i1p1) for each CMIP5 model, which limits the internal climate variability probed by this analysis. This work only examined changes in summer wildfire burn area. We did not consider how the length of the wildfire season is likely to increase (Yue et al., 2013), or how that could impact future burn area. Our work shows that changes to environmental variables in the RCP 8.5 scenario result in larger wildfire potential than RCP 4.5. Other work has demonstrated that RCP 4.5 could lead to episodically larger wildfires in the future as that pathway has conditions conducive to fuel build up (Bachelet et al., 2016). Our methods do not account for feedbacks like this. Finally, our analysis is based on only three seasons: the prior winter (months November, December, January, and February), the prior spring (months March, April, and May), and summer (months June, July, and August). This allows for short‐term (i.e., within 1 year) lagging relationships between environmental variables and summer burn area, in fuel‐limited regions moisture availability in the year or 2 years prior can promote fire via fuel growth (e.g., Abatzoglou & Kolden, 2013; Littell et al., 2009; Swetnam & Betancourt, 1998; Williams et al., 2015, 2019).

Despite these shortcomings, this work offers novel contributions. We show how historical relationships between environmental conditions and changes to those conditions simulated by CMIP5 may contribute to future burn area for multiple western U.S. ecoregions. We select these variables objectively based on those that best explain historical wildfire variability. This work shows that estimates of future burn area are largest whenever and wherever the importance of VPD is high. We show that increases in burn area in the western United States are likely, as changes in VPD, as well as other candidate variables, indicate the future will likely be drier. CMIP5 models are in agreement that VPD will increase in the 21st century, largely driven by increasing temperatures. VPD is the only variable that increases across all ecoregions, models, and RCPs, thus using VPD as a key predictor of future burn area will likely underestimate uncertainty.

## Conflict of Interest

The authors declare that they have no conflicts of interest.

## Supporting information

Supporting Information S1

## Data Availability

All data used to make the figures and conclusions presented in this manuscript are publicly available. The links to these data are provided in section 2. In addition, all codes used in this analysis are available at the following url https://zenodo.org/record/3872600#.XtXQ2p5KgWo (DOI: 10.5281/zenodo.3872600).
