# Supplementary material for: Past Variance and Future Projections of the Environmental Conditions Driving Western U.S. Summertime Wildfire Burn Area
Source: Earths Future. 2021 Feb 6;9(2):e2020EF001645. doi: 10.1029/2020EF001645 (PMC7900977; doi:10.1029/2020EF001645)
Supplement: Supplementary file 1 — Supporting Information S1 [file EFT2-9-e2020EF001645-s001.docx]

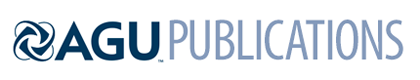


*[Earths Future]*

Supporting Information for

**Examining the future spread in environmental conditions that drive western U.S. summertime wildfire burn area**

Steven J. Brey^1^,  Elizabeth A. Barnes^1^,  Jeffrey R. Pierce^1^, Abigail L.S. Swann^2^, and Emily V. Fischer^1^

^1^Colorado State University Department of Atmospheric Science, Fort Collins, Colorado, USA

^2^University of Washington Department of Atmospheric Science, Seattle, Washington, USA

**Contents of this file**

Figures S1

**Introduction**

This supporting information contains a figure referenced in the main manuscript. This figure shows the linear model coefficient sensitivity to leaving out individual years of predictor and burn area data for the fits.


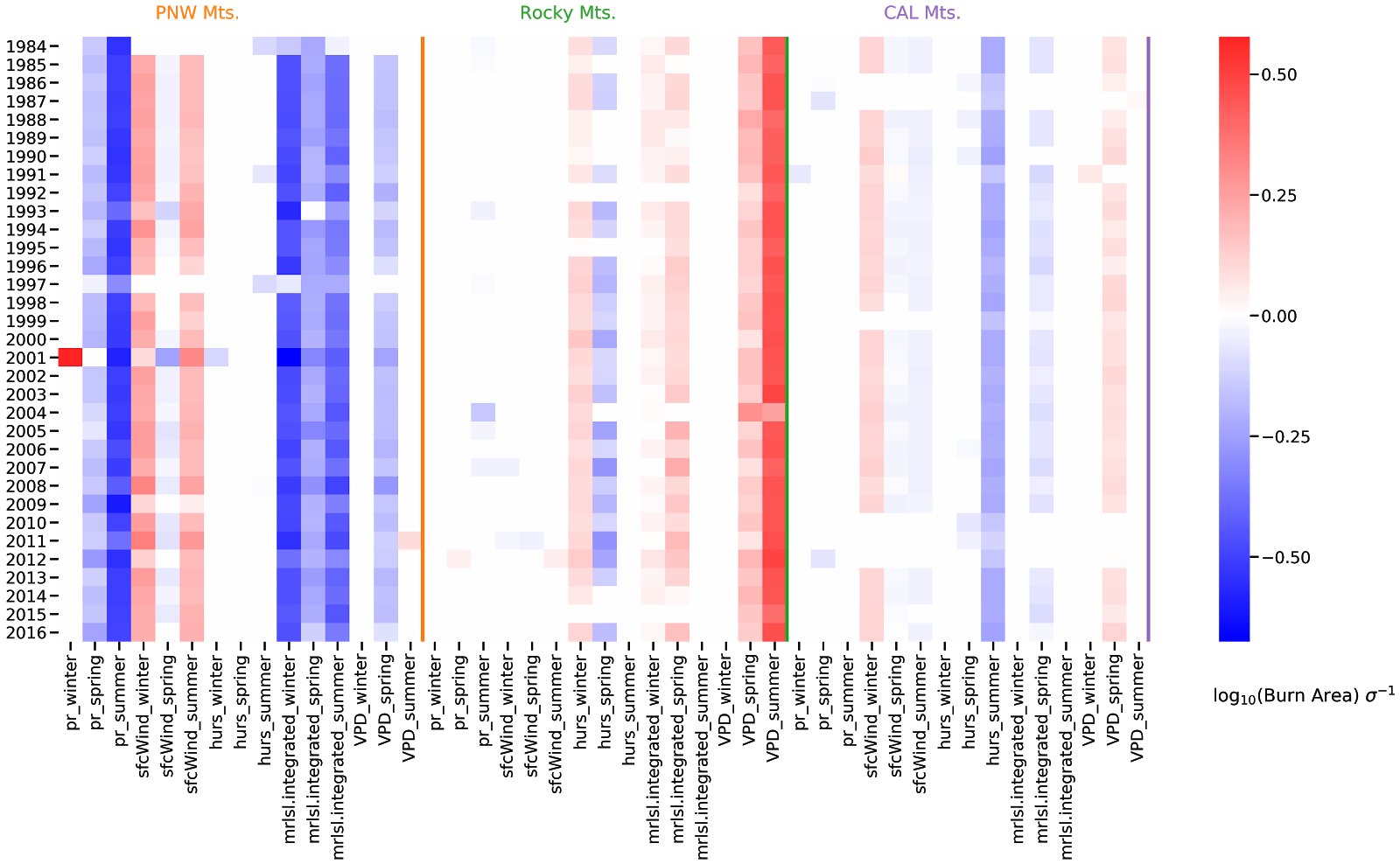


**Figure S1:** Each row shows the value of the coefficients (color) for different Lasso regression models fit in each of the three ecoregions in this work (separated by vertical colored lines). The vertical axis shows the year that was not included in fitting each Lasso regression model. Each Lasso regression model was fit after tuning alpha (regularization term) via leave-one-out (year) cross validation. Ecoregions are denoted and separated by the colored vertical lines. Combined with Figure 2 in the manuscript, these figures provide a comprehensive overview of how sensitive regression model variable coefficients are to what data are used.
